# Hospital Needs

**Published:** 1912-11-16

**Authors:** 


### Hospital Needs.

### LACTAGOL.

(E. T. Pearson and Co., Ltd., 49 Watling Street, E.C.)

This substance is a powdered extract of cotton seed, and is stated to exercise a specific action on the mammary gland. According to the evidence of some who have tried it, lactagol increases the mammary secretion of the nursing mother, both in quantity and in quality. Lactagol is given in milk, cocoa, coffee, gruel, or soup. If taken at once after being prepared it imparts, we find, practically no alien taste to those vehicles, but a slightly gritty sensation is apparent to the tongue. If allowed to stand for some time, this grittiness disappears, and then a faint taste is perceptible which is not actually disagreeable, but at the same time is not an improvement. The substance is stated to be insoluble in water, but this observation would seem to show that some part of it at least is diffusible. We have so far had opportunities of testing lactagol on one patient only. This was an otherwise normal healthy primipara, in whom lactation was almost nil, in spite of the most persevering efforts on her part. A week's administration of lactagol was followed by a slight improvement in the milk, both as to quality and quantity; but as the amount was still nowhere near sufficient for the needs of the infant, the attempt at breast-feeding was abandoned, and hand-feeding resorted to. So far, therefore, as this small experience goes, it bears out the claims made for lactagol; but it is also evident that the specific action, if such there be, is limited in its range, and that expectations must not be raised too high. We hope doctors and nurses will give it a fair trial, and that the results will prove to be satisfactory both to them and to their patients.

### THE "EUREKA" CREPE VELPEAU BANDAGE.

This bandage has received already encomiums in The Hospital, which was able to give very high praise to a specimen submitted for judgment two years ago. It was then pointed out that the "Eureka" Crêpé Velpeau Bandage contained no rubber, and that its elastic, cool, and porous qualities made it very easily washable, and therefore economical and lasting. For the treatment of varicose veins this bandage has been found to be far superior to ordinary qualities, which, it was pointed out, lose their elasticity in spite of the high price charged for them. Lightness, porousness, and ready adjustability are the valuable qualities of this Velpeau Bandage, which can be obtained from Mr. Vincent Wood, surgical appliance maker, Victoria House, Albion Place, Blackfriars Bridge, S.E.

### A GOLD MEDAL HOT-WATER BOTTLE.

For hospital purposes the rubber hot-water bottle has practically replaced the earlier stone variety, but discrimination is still required in the choice of materials and design. Mr. Vincent Wood, surgical appliance maker, Victoria House, Albion Place, Blackfriars Bridge, S.E., has placed on the market in the "Eureka" Hot-water Bottle, with its special bag cover, a type that is its own recommendation. In 1908, it is recorded, the firm's bottles won a gold medal at the Franco-British Exhibition. Guaranteed as British made and thoroughly reliable, the prices vary with the size from 2s. 8d. (8 by 6) through nine intermediate sizes to 7s. (16 by 12), and of course special prices are quoted for large quantities by weight. A word as to the covers should be added. The new "Eureka" Bag Cover, in plush or pure lambs' wool, has a special advantage in that the whole bottle is covered, including the handle, so that no cold part can touch the patient. The inclusive price for this cover is 11s. per dozen in plush and 11s. 6d. in lambs' wool.

### THE SURGICAL MANUFACTURING COMPANY.

A new Company has been formed under the title of The Surgical Manufacturing Company, at 85 Mortimer Street, W. The Company, which is under the control of Mr. W. J. Edwards, late managing director of the Hospitals and General Contracts Company, is a combination of manufacturers; and, as there will be no intermediate profits, it will be in a position to supply surgical and nursing articles at the minimum price. Its showrooms will contain the latest appliances for nursing the sick and injured. A speciality will be made of hiring out invalid furniture and appliances, water-beds, etc., and all money paid for hire will go towards the purchase of the article at list price. Moreover, the Company will send out on hire complete outfits for septic operations in private houses, consisting of operating table, instrument table, steriliser, jugs, trays, sterilised towels, overalls, and every requisite. The Company's illustrated catalogue and nurses' case-book will be sent post free on application.

